# Behavioral, cortical and autonomic effects of single-dose escitalopram on the induction and regulation of fear and disgust: Comparison with single-session psychological emotion regulation with reappraisal

**DOI:** 10.3389/fpsyt.2022.988893

**Published:** 2023-01-04

**Authors:** Myrto Sklivanioti Greenfield, Yanlu Wang, Mussie Msghina

**Affiliations:** ^1^Department of Clinical Neuroscience (CNS), Karolinska Institute, Stockholm, Sweden; ^2^Department of Clinical Science, Intervention, and Technology, Karolinska Institute, Stockholm, Sweden; ^3^MR Physics, Medical Radiation Physics and Nuclear Medicine, Karolinska University Hospital, Stockholm, Sweden; ^4^Department of Psychiatry, Faculty of Medicine and Health, Örebro University, Örebro, Sweden

**Keywords:** fNIRS, EDA, fear, disgust, emotion induction, emotion regulation, escitalopram, SSRI

## Abstract

**Introduction:**

Adaptive and successful emotion regulation, the ability to flexibly exert voluntary control over emotional experience and the ensuing behavior, is vital for optimal daily functioning and good mental health. In clinical settings, pharmacological and psychological interventions are widely employed to modify pathological emotion processing and ameliorate its deleterious consequences.

**Methods:**

In this study, we investigated the acute effects of single-dose escitalopram on the induction and regulation of fear and disgust in healthy subjects. Furthermore, we compared these pharmacological effects with psychological emotion regulation that utilized a cognitive strategy with reappraisal. Emotion induction and regulation tasks were performed before and 4 h after ingestion of placebo or 10 mg escitalopram in a randomized, double-blind design. The International Affective Picture System (IAPS) was used as a source of images, with threat-related pictures selected for fear and disease and contamination-related pictures for disgust. Behavioral data, electrodermal activity (EDA), and functional near-infrared spectroscopy (fNIRS) recordings were collected.

**Results:**

Escitalopram significantly reduced emotion intensity for both fear and disgust during emotion induction, albeit with differing electrodermal and hemodynamic activity patterns for the two negative emotions. At rest, i.e., in the absence of emotive stimuli, escitalopram increased sympathetic activity during the fear but not during the disgust experiments. For both fear and disgust, emotion regulation with reappraisal was more effective in reducing emotion intensity compared to pharmacological intervention with escitalopram or placebo.

**Discussion:**

We concluded that emotion regulation with reappraisal and acute administration of escitalopram, but not placebo, reduce emotion intensity for both fear and disgust, with cognitive regulation being significantly more efficient compared to pharmacological regulation under the conditions of this study. Results from the fNIRS and EDA recordings support the concept of differential mechanisms of emotion regulation that could be emotion-specific.

## 1 Introduction

Flexible and adaptive emotion management is fundamental for optimal daily functioning and good mental health, while impairments in emotional processing, such as the perception, induction and regulation of emotions, are known to be core features of various psychiatric ailments, particularly affective and anxiety disorders (1–3). Although the very nature of emotions is still widely debated (4), specific emotions are assumed to make up core symptoms of psychiatric disorders such as depression (sadness), anxiety (fear) and obsessive-compulsive disorder (disgust) (5–9). In clinical settings, pharmacological and psychological interventions are widely employed to modify pathological emotion processing and ameliorate its deleterious consequences on mental health.

Emotion regulation utilizes a variety of strategies including reappraisal (cognitive re-evaluation of the significance of the stimulus/situation at hand) and suppression (10–16). Within the scope of cognitive control, emotion regulation is of particular interest because of its clinical relevance, its association with a myriad of psychiatric diagnoses, and of it being the main target of various forms of psychological therapy methods, including those that utilize reappraisal strategies (17–20). Previous research has shown that reappraisal is more effective, at least for moderate intensity emotions, and is associated with better cognitive and social outcomes compared to suppression (21). In the clinical setting, psychological treatment particularly that which utilizes cognitive strategy is comparable to pharmacological intervention for mild to moderate depression and anxiety disorders (3, 22, 23).

Serotonin is an important neuromodulator, implicated in various cognitive and affective processes, including the induction, perception and regulation of emotions (24). Also, although there is evidence pointing otherwise and the role of serotonin in affective disorders is by no means undisputed (25), aberrant serotonergic transmission has been linked to psychiatric disorders, including major depression and anxiety disorders (26, 27). Correspondingly, pharmacological treatment of depression and anxiety disorders involve serotonergic agents, and selective serotonin reuptake inhibitors (SSRI) are first-line treatment options in most cases (28). Escitalopram, an antidepressant of the SSRI class, blocks the serotonin transporter in axon terminals increasing serotonin levels in the synaptic cleft (29), although the net effect on serotonin concentrations in the projection areas is also mitigated by activation of 5-HT auto-receptors (30–34). Patients treated with SSRIs generally respond within 4-12 weeks after the start of treatment (35), but a substantial number report increased anxiety and blunted emotions as early side effect of treatment (36). Especially children and young adults run increased risks of negative consequences of these early side effects, which in some cases may lead to dysregulated emotions and increased suicidal ideation during the initiation of SSRI treatment (37).

Besides their broad clinical application, SSRIs in conjunction with brain imaging are also frequently used as pharmacological probes to gauge the role of serotonin in emotion induction and regulation. Hemodynamic activity of prefrontal cortex (PFC) and amygdala has thus been shown to be affected by changes in the serotonergic transmission (38–40), but the direction of modulation seems to vary with task and brain region and both increased and decreased activations have been reported (32, 41). For example, hemodynamic activity in amygdala has been found to increase or decrease during facial emotion recognition tasks after ingestion of SSRI (42–46). Similarly, there are reports of increased and decreased activations of PFC areas after intake of SSRI (30, 43, 46–48). More specifically, single dose SSRI was associated with increased startle responses (32, 49–51), and enhanced detection of facial expressions of fear and happiness without affecting that of anger, sadness and disgust (52). Murphy, Norbury (42) reported that single dose SSRI decreased recognition of disgust and amygdala response to fear, while Outhred, Das (53) using escitalopram reported reduced activation of right inferior frontal gyrus during emotion induction (54). In another study, Outhred, Das (55) investigated the acute effects of escitalopram on emotion regulation with reappraisal and concluded that escitalopram facilitated regulation of negative emotions few hours after its ingestion, while clinical experience and findings from other studies seem to indicate that SSRIs, at least initially and especially among young adults, may be more prone to cause emotional dysregulation (37).

Studies investigating psychological regulation of emotions have also demonstrated changes in the fronto-limbic circuits. Lateral PFC, especially dorsolateral areas, have been associated with cognitive control of behavior (56–59), and its activation changes in tandem with cognitive regulation of emotion with reappraisal (60, 61). Long-term psychological treatments that promote cognitive reappraisal skills (3, 62, 63), and successful psychotherapy improving cognitive control have also been associated with post-treatment reduction in PFC activity (64–66).

In the present study, we investigated the effects of single-dose escitalopram on emotional processing using behavioral measures, functional near infrared spectroscopy (fNIRS) and electrodermal activity (EDA). fNIRS is a non-invasive imaging method that allows the estimation of relative changes in the concentration of oxygenated (oxy-Hb) and deoxygenated hemoglobin (deoxy-Hb) and has been found to be well correlated with functional magnetic resonance imaging (67, 68). Despite the fact that fNIRS detects changes in hemodynamic activity only in the cortical mantle, with deeper brain areas left inaccessible, a multimodal approach combining central, peripheral, and subjective measures can provide a broad picture of the emotional processes of interest (69–73). EDA measures changes in sympathetic nerve activity and is often used as a peripheral marker of cognitive and affective processes related to limbic and PFC activity (74). Although not specific for a given emotion by itself (75), previous studies have shown physiological differences in the responses different emotions preferentially elicit; for example, sympathetic activation for fear and parasympathetic activation for disgust (8, 61, 76, 77).

Based on previous literature and clinical experience with patients initiated on SSRI treatment, we hypothesized that acute ingestion of 10 mg escitalopram would (i) increase anxiety and resting sympathetic activity in the absence of emotive stimuli, (ii) blunt emotional reactivity in response to emotive stimuli, and (iii) hamper cognitive control of emotion leading to emotional dysregulation. Lastly, it is not known whether single-session pharmacological and psychological modulations of emotions are equally effective in downregulating negative emotions, and whether they utilize similar or different underlying neurobiological mechanisms. We compared therefore the effects of single-dose pharmacological regulation with escitalopram with single-session psychological regulation with reappraisal of the two negative emotions fear and disgust.

## 2 Materials and methods

### 2.1 Participants

The study subjects [*n* = 26 in the escitalopram arm (54 % females, mean age 32.1 ± 9.7 years) and *n* = 20 in the placebo arm (60 % females, mean age 31.8 ± 9.2 years)] were recruited from non-clinical population by advertisement in Psychiatry Southwest and Karolinska University Hospital, Huddinge, Sweden. The sample size was calculated after performing a power analysis based on a pilot fNIRS study and previous similar fMRI studies (78), see also Supplementary material. Prior to the first trial, an overview of the general scope of the study and the outline of the experimental procedure were given. All subjects met the following inclusion criteria: able and willing to provide written informed consent, >18 years of age or older at the time of recruitment, free of any psychiatric, neurological and addiction disorders, as well as any current drug use including psychoactive medication. All subjects were asked to abstain from alcohol consumption at least one day prior to the trial and were instructed to continue their usual consumption of coffee and nicotine and keep it the same level prior to each part of the testing.

### 2.2 Ethics

The study was approved by the Stockholm County’s ethics committee (Dnr 2013/722-32, 2014/436-32, and 2022-02605-02). All subjects were given verbal and written information and gave written informed consent through their signature prior to the start of the experiment, in accordance with the Declaration of Helsinki.

### 2.3 Study design

#### 2.3.1 Experimental design

The experimental setup included a counterbalanced block design with a randomized order of sequence of the two tasks (*fear* and *disgust*), examining the induction and regulation of the two negative emotions.

**FIGURE 1:**
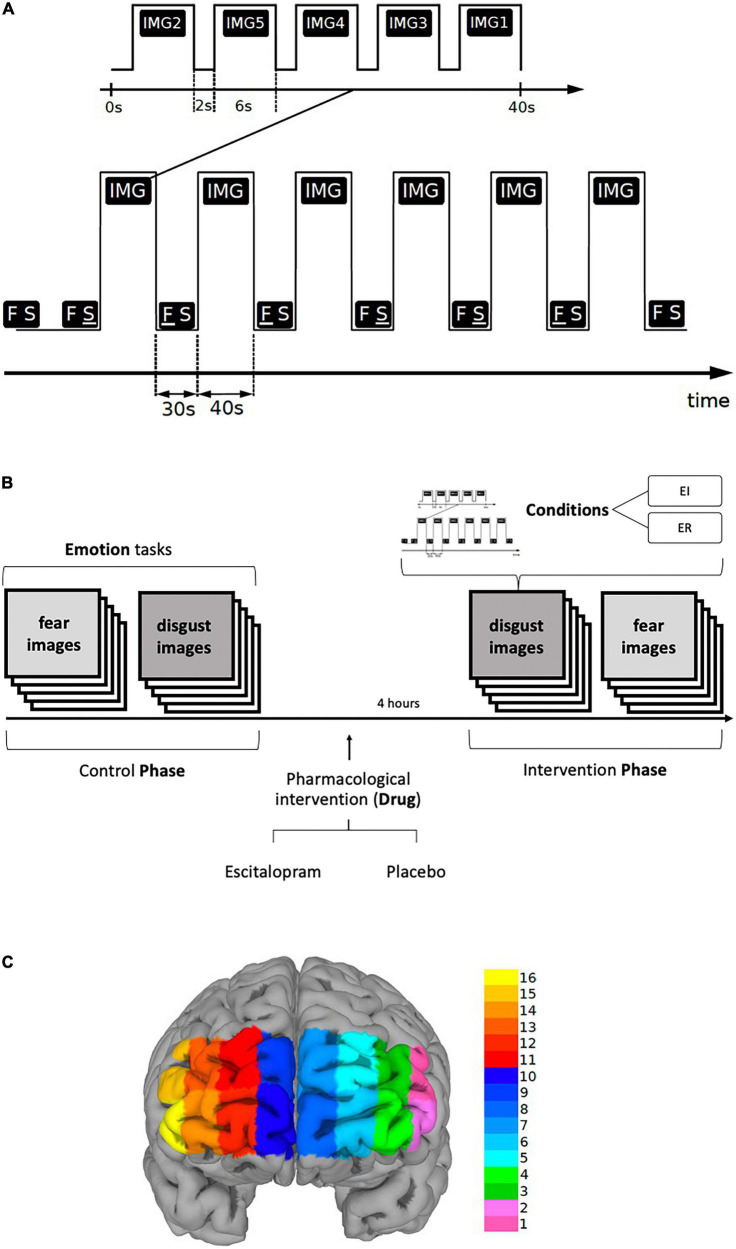
**(A,B)** Paradigm timing for each task (fear and disgust). Each task included six blocks of stimuli (IMG), each block was 40-s long and was preceded by a 30-s long period of REST (FS). In each block, five different visual emotive stimuli (pictures IMG1-5) were presented on the screen, each for six seconds, followed by a two-seconds long interval to separate them from each other. Immediately after viewing the pictures, the subjects were asked to score emotion intensity for every image in a scale ranging from 1 to 9 during emotion induction (EI) and emotion regulation (ER). The identical procedure was repeated four hours after the ingestion of escitalopram or placebo (intervention phase). The order of the images, conditions and tasks was randomized. **(C)** Shows the fNIRS channel locations on the PFC. The data were averaged over left (LPFC, channels 1-6), medial (MPFC, channels 7-10) and right (RPFC, channels 11-16) prefrontal regions to increase signal-to-noise ratio.

Each task included six blocks of stimuli, each block was 40-s long and was preceded and followed by a 30-s long period of *Rest*. In each block, five different stimuli, i.e., pictures representative for respective emotion, were presented, each for six seconds, followed by a two-seconds long interval to separate them from each other (Figures 1A, B).

The six blocks were randomly assigned in a counterbalanced way to one of the two conditions, *Emotion Induction (EI)* or *Emotion Regulation (ER)*, each condition being repeated three times thus allowing the functional response to be disentangled from physiological confounds. During *Rest*, the screen displayed two letters “F S” on a white background, the letter F underlined (F S) denoting the ensuing pictures as an EI block, whereas when the letter S was underlined (F S) the succeeding block was denoted as an ER block. Participants were told that when the letter “F” is underlined to simply look at the pictures allowing them to induce any emotional response, but when the letter “S” is underlined to try to reduce the intensity of emotion the pictures generated by reevaluating their significance. In addition, they were given examples of reappraisal and were instructed not to use other emotion regulation methods, such as suppression. To reduce carry-on and anticipatory effects, the order of conditions as well as the order of the stimuli within each block were randomized. The tasks were implemented in E-Prime (version 2.1)^1^. Primary and secondary outcomes were defined for the behavioral, fNIRS and EDA measures. For behavioral data, we used emotion induction and emotion regulation scores as primary outcomes, and emotion regulation index, “pharmacological” and “psychological,” as secondary outcomes. The fNIRS analysis was based on region-wise analysis of left (LPFC), right (RPFC) and medial prefrontal cortex (MPFC) for EI and ER activations. Lastly, for EDA phasic electrodermal response (EDR) frequency was defined as the primary outcome (see also data acquisition section and Supplementary material).

#### 2.3.2 Emotion induction and emotion regulation tasks

The International Affective Picture System [IAPS, (79)] was used as a source of the standardized pictures for the tasks. Threat and physical harm related images were selected for fear and disease and contamination related images for disgust. Additionally, the images were explicitly labeled in each block as representing fear or disgust, respectively (see Supplementary material for details and id number of the selected images for each emotion). As described above, the participants were instructed to either passively view the ensuing pictures (emotion induction, EI) or to actively down-regulate the emotion using reappraisal (emotion regulation, ER). To ascertain that they conformed to the instructions given to them, the participants were interviewed immediately after the end of the experiment as to the specific strategy they used, which was documented verbatim. To limit the effect of confounds, such as temperature and humidity, that can affect measurements, experiments were conducted in a closed room with dimmed lighting, stable and regulated temperature and humidity and measurement was started several minutes after participants had stayed in the room connected to the fNIRS device and reported feeling relaxed and comfortable.

#### 2.3.3 Pharmacological intervention

Participants arrived in the morning, performed the experiment without pharmacological intervention (*Control Phase*), and ingested either 10 mg escitalopram or placebo in a blinded fashion (intervention). Approximately four hours later, participants returned to perform the same experimental procedure again (*Intervention Phase*) (Figure 1B). The intervention consisted of 10 mg escitalopram in the form of Cipralex^®^ oral drops mixed in a glass of water (350 ml) or placebo (same amount of water with no escitalopram added to it). To control for blinding, the participants were asked to hazard a guess as to which of the interventions they thought they had received at the end of the experiment. They were also asked to freely state any side effect they experienced.

### 2.4 Data acquisition and analysis

#### 2.4.1 Behavior measures of subjective emotion experience

At the end of the experiments, immediately after viewing the images, the participants were asked to score emotion intensity for every image in a scale ranging from 1 to 9 during EI and ER, where 1 represented lowest and 9 highest level of emotion intensity. In specific they were asked to rate the “intensity of the experienced (induced or regulated) emotion in response to the image.” The mean score for the images was calculated to represent emotion rating for each condition (EI and ER).

Emotion regulation using reappraisal, here termed “Psychological ER,” was calculated based on the rating for emotion induction (EI) and emotion regulation (ER) using the following formula:


$$\begin{array}{l} Psychological\;Emotion\;Regulation\;Index\;\left(Psych\;ER\;index\right)\\ =\frac{\left(EI\;in\;Control\;–\;ER\;in\;Control\right)}{EI\;in\;Control} \end{array}$$

Emotion regulation as a result of pharmacological intervention with escitalopram or placebo, here named ‘Pharmacological ER’, was calculated based on the rating for emotion induction (EI) before and after ingestion of escitalopram or placebo using the following formula:


$$\begin{array}{l} Pharmacological\;Emotion\;Regulation\;Index\;(Pharm\;ER\;index)\\ =\frac{\left(EI\;in\;Control\;–\;EI\;after\;Intervention\;\left(esc\;or\;placebo\right)\right)}{EI\;in\;Control} \end{array}$$

#### 2.4.2 Functional near-infrared spectroscopy (fNIRS) recordings

A continuous wave fNIRS device consisting of a flexible headband holding light sources and detectors (fixed distributions), and a fNIR100 data acquisition box with a sampling rate of 2 Hz connected to a personal computer via an MP150 data acquisition and analysis system (Biopac Systems Inc., JOR AB Sweden) was used to measure the relative changes in the concentration of oxy-hemoglobin (Δoxy-Hb). The headband was placed on the forehead of the participant and the sensor consisted of four infrared light sources emitting at two different wavelengths (730 and 850 nm) and ten detectors separated by a distance of 2.5 cm, giving a total of 16 channels for recording different parts of the prefrontal cortical mantle (mainly BA 9, 10, 45, 46 (80)) as shown in Figure 1C. Electrode placement was done according to the protocol recommended by Biopac Systems Inc., and as described by Ayaz and colleagues (81) Ayaz, Onaral (80). Participants were asked to lift their hair from the forehead before sensor placement, the sensor strip was placed just above the eyebrows and the center of the sensor was matched with the vertical axis of symmetry that passes through the nose. Data acquisition was performed using the COBI Studio software (Cognitive Optical Brain Imaging Studio, fNIRS Devices LLC) and a second personal computer was connected to the system via a COM cable to synchronize the E-Prime data set with the fNIRS and EDA data sets using Acqknowledge software version 4.2 (Biopac Systems Inc, JOR AB Sweden). Raw data was converted to levels of oxygenated (oxy-Hb) and deoxygenated hemoglobin (deoxy-Hb) by COBI software utilizing a modified Beer-Lambert Law.

##### 2.4.2.1 Preprocessing and statistical analysis

For the fNIRS data we used “NIRS-SPM toolbox” (82) that utilizes the SPM12 package (Wellcome Department of Cognitive Neurology, London, UK) and runs under MATLAB (MATLAB_R2019b, Mathworks, Natick, MA). For the present study, we chose to analyze oxy-Hb, because it measures more reliably task-related activation (72, 83–86) and has better signal-noise ratio (67, 87). Physiological noise including artifacts from respiration and cardiac pulsation was removed using two band-stop filters (0.12-0.25 and 0.7-2.0 Hz). Less than 5% of the channels (without specific channels being overrepresented) in all trials were excluded because of technical quality problems. For detrending and reducing low-frequency confounders, a high-pass filter based on a discrete cosine transform set with the cut-off period set to 128 s was utilized. Autocorrelations in the time series due to hemoglobin changes were corrected using the pre-whitening method from the NIRS-SPM toolbox (88).

Using generalized linear model (GLM), the data from each channel was separately fitted to the ideal responses modeled through the onset timings with the hemodynamic response function consisting of the canonical HRF and its temporal and dispersion derivatives. Two t-contrasts were calculated for each channel: (1) Emotion Induction vs. Rest [EI – REST] and (2) Emotion Regulation vs. Rest [ER – REST]. Channel-specific beta coefficients were generated, which were then used for further statistical analyses. The data were averaged over left (LPFC, Channels 1-6), medial (MPFC, Channels 7-10) and right (RPFC, Channels 11-16) prefrontal regions to increase signal-to-noise ratio. Outlier correction was performed by replacing outliers with the Q1 – 1.5 IQR and Q3 + 1.5 IQR rather than outright removing them, as a more conservative approach.

### 2.5 Electrodermal activity (EDA)

For the measurement of EDA as skin conductance, two non-polarizable Ag-AgCl electrodes (EL 507, JOR AB Sweden) were placed on the middle phalanges of digits 2 and 3 of the left hand (exosomatic recordings using a direct current) to record electrodermal activity (EDA) using GSR100C amplifier of the Biopac MP150 system and captured with Acqknowledge software version 4.2 (Biopac Systems Inc, JOR AB Sweden). Amplifier gain was set at 10 μmho/V, low-pass filter at 1 Hz and high-pass filter at 0.05 Hz. After data acquisition, to remove artifacts and high-frequency noise, a low-pass filter (5th-order low-pass Butterworth filter with cut-off frequency at 1 Hz) and a median smoothing (smoothing window equal to the sample frequency 8 Hz) were applied to the raw EDA signals. Subsequently, the pre-processed signal was decomposed into three components: tonic signal, phasic signal and white Gaussian noise using a convex optimization approach (89). Lastly, using Matlab EDA Toolbox^2^, we calculated the frequency of phasic electrodermal responses (EDR), for the average of the seven Rest periods (spontaneous non-specific electrodermal responses – “NS-EDR”), and for the three EI and three ER periods, respectively (stimulus-evoked EDR, here abbreviated as “EDR”). The latency windows for EDR onset were set to 1–3 s after stimulus onset.

### 2.6 Data analysis

We used a block-design to assess differences between tasks, phases and conditions. A set of multilevel mixed-effects linear regression models (fixed effects: EMOTION (fear and disgust) x CONDITION (emotion induction and emotion regulation) x PHASE (control and intervention) x DRUG (placebo and escitalopram), random effects: intercepts for subjects due to repeated measures; method of estimation: maximum likelihood) were applied to the dependent measures of primary outcomes (behavioral measures, fNIRS and EDA data) and the Benjamini-Hochberg method used to control for multiple testing (raw *p*-values are reported). Two-tailed t-tests, with the probability of rejecting the null hypothesis set at *p* < 0.05 (adjusted according to the Bonferroni correction method), were subsequently performed to explore significant contrasts in comparison to rest or baseline conditions for fNIRS and EDA as well as for the secondary analyses. Normality was tested and non-parametric tests were performed where relevant. All statistical analysis was performed using Stata 14 software (StataCorp. 2015. Stata Statistical Software: Release 14. College Station, TX: StataCorp LP).

## 3 Results

In the following sections, we use the term ‘control’ to refer to the absence of ingested drugs (namely the experimental phase *before* the intervention phase where escitalopram or placebo were given) and the term “rest” to refer to the absence of emotive stimuli (namely the periods in the experiment without blocks of stimuli).

### 3.1 Behavioral data

#### 3.1.1 Emotion induction

**FIGURE 2:**
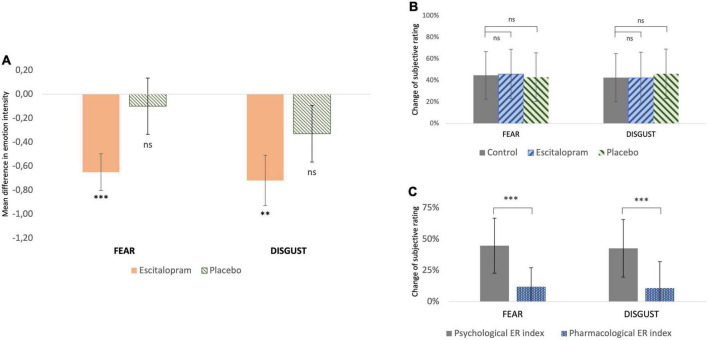
Behavioral measures **(A)** change of subjective rating of emotion intensity from baseline (control) after pharmacological intervention with escitalopram and placebo, respectively. For both fear and disgust, escitalopram significantly reduced emotion induction, whereas placebo had no significant effect (shown mean difference and SE in score in a scale 1-9, significance asterisk declare significance related to 0). **(B)** Change of subjective rating of emotion intensity while applying reappraisal before (control) and after pharmacological intervention with escitalopram and placebo, respectively. **(C)** Effortful emotion regulation with reappraisal (operationalized as psychological ER index) was associated with a mean decrease of ca 45% in emotion induction of both fear and disgust. This reduction was significantly larger than the one associated with one dose escitalopram (operationalized as pharmacological ER index) under the conditions of this study.

Compared to control, emotion intensity during emotion induction (EI) was significantly reduced after ingestion of 10 mg escitalopram for both fear (contrast –0.70 ± 0.28, *p* = 0.011; mean effect –11.9% ± 15.1%, *p* < 0.001) and disgust (contrast –0.68 ± 0.28, *p* = 0.016; mean effect –10.8% ± 21.1%, *p* = 0.02), with no significant difference in this between the two negative emotions (mean difference between fear and disgust 2% ± 3%, *p* = 0.6). Placebo had no significant effect on fear (*p* = 0.67) or disgust (*p* = 0.18) (Figure 2A).

#### 3.1.2 Emotion regulation

For both fear and disgust, subjects rated emotion intensity higher when they simply attended to the pictures (EI), compared to when they cognitively downregulated it using reappraisal (ER). Under these conditions, emotion intensity decreased by roughly 40-45% (*p* < 0.001), in an effect here termed as “psychological emotion regulation.” Neither escitalopram nor placebo had any significant effect on the efficacy of psychological emotion regulation with reappraisal for fear (*p* = 0.52 and *p* = 0.73 for escitalopram and placebo, respectively) or disgust (*p* = 0.86 and *p* = 0.98 for escitalopram and placebo, respectively) (Figure 2B). When psychological emotion regulation (reappraisal) was directly compared with pharmacological emotion regulation (escitalopram), reappraisal was found to be significantly more effective than escitalopram in reducing emotion intensity for fear (contrast −1.77 ± 0.33, *p* < 0.001; mean difference 32.7% ± 28.5%, *p* < 0.001) and disgust (contrast −1.85 ± 0.28, *p* < 0.001, mean difference 31.8% ± 30.3%, *p* < 0.001) (Figure 2C).

### 3.2 Functional near-infrared spectroscopy recordings (fNIRS)

#### 3.2.1 Emotion induction

**FIGURE 3:**
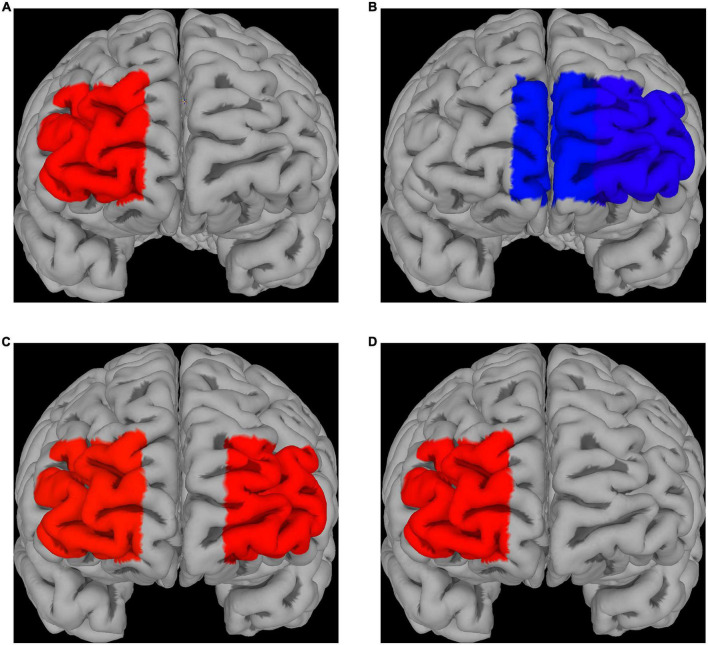
fNIRS recordings. Differences in Δoxy-Hb levels during emotion induction (EI) [EI – REST] for fear **(A)** and disgust **(B)**; significantly greater activations compared to the control phase were seen in right PFC for fear after escitalopram was administered, whereas significantly less activation in medial and lateral PFC was seen in disgust. **(C)** Differences in Δoxy-Hb levels during EI [EI – REST] when comparing the two emotions after escitalopram administration show greater activation of lateral PFC in fear compared to disgust. **(D)** Direct contrast psychological emotion regulation (reappraisal) [ER contrast in control] with pharmacological emotion regulation (escitalopram) [EI contrast in Intervention phase, escitalopram] in fear demonstrated greater activation for the latter in right PFC, with no PFC areas found to be significantly more activated by reappraisal compared to escitalopram. No significant differences were found for disgust.

Compared to control (absence of ingested drugs), placebo had no significant effect on fNIRS activations for both fear and disgust, while escitalopram increased fNIRS activations in right lateral prefrontal cortex (PFC) for fear (contrast 0.58 ± 0.26, *p* = 0.025) and decreased it in left lateral (contrast −0.55 ± 0.26, *p* = 0.032) and left medial PFC for disgust (contrast −0.47 ± 0.24, *p* = 0.05; Figures 3A, B). When the two negative emotions were directly compared to each other, there was no significant differences in the placebo group, but the escitalopram group had significantly greater activation for fear compared to disgust in right (contrast 0.62 ± 0.26, *p* = 0.015) and left lateral PFC (contrast 0.60 ± 0.26, *p* = 0.02) (Figure 3C).

#### 3.2.2 Emotion regulation

Neither placebo, nor escitalopram had any significant effect on PFC activations during emotion regulation for any of the two negative emotions. When psychological emotion regulation (reappraisal) was directly compared with pharmacological emotion regulation (escitalopram), we found significantly greater activation during escitalopram for fear in right PFC (contrast 0.58 ± 0.26, *p* = 0.026), with no PFC areas more active during reappraisal compared to escitalopram (Figure 3D). For disgust, there were no significant differences in activation when psychological and pharmacological regulations were compared to each other.

### 3.3 Electrodermal activity (EDA)

#### 3.3.1 Resting conditions in the absence of emotive stimuli

**FIGURE 4:**
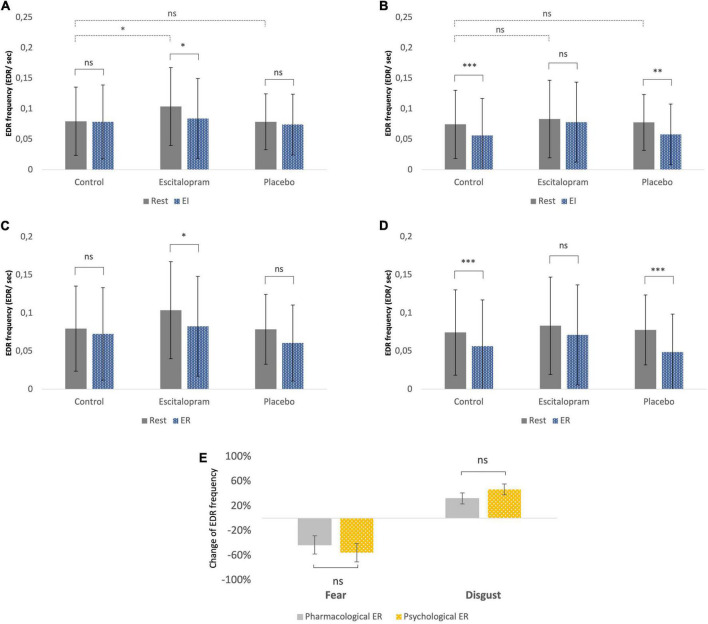
Autonomic measures (electrodermal activity – EDA frequency). **(A)** Fear, emotion induction. **(B)** Disgust, emotion induction. **(C)** Fear, emotion regulation. **(D)** Disgust, emotion regulation. The EDR labeling in the *Y*-axon includes both spontaneous NS-EDR (Rest columns) and stimulus-evoked EDR (EI and ER columns). **(A,B)** The dotted line comparisons in A (fear) and B (disgust) show that compared to control phase, after ingestion of escitalopram, a roughly 30% increase in electrodermal response frequency was measured at rest (absence of emotive stimuli) during the fear experiments but not the disgust ones. **(A–D)** The solid line comparisons show that, similarly to what was previously reported in a study without pharmacological intervention (61), placebo was associated with no difference between task EDA and rest in fear **(A,C)** but lower than rest in disgust **(B,D)**. This pattern was reversed in the escitalopram arm, where task EDA was significantly lower than rest in fear **(A,C)** but not significantly different than rest in disgust **(B,D)**. Shown mean EDA frequency, EDR per second, and SD. **(E)** Emotion regulation with reappraisal and escitalopram did not differ significantly regarding the effects on EDA. Shown mean change in EDA frequency (EDR per second) and SE of this difference. ***p < 0.001, **p < 0.01, and *p < 0.05. Pharmacological ER = (EDA EI in control – EDA EI in intervention phase)/EDA EI in control. Psychological ER = (EDA EI in control – EDA ER in control phase)/EDA EI in control (all adjusted to baseline/EDA rest).

In the absence of emotive stimuli (rest), escitalopram increased non-specific spontaneous phasic electrodermal response (NS-EDR) frequency by roughly 30% during the fear (mean difference 0.03 ± 0.01 Hz, *p* = 0.008), but not disgust experiments (*p* = 0.95). Placebo had no significant effect on NS-EDR frequency (*p* = 0.4 and *p* = 0.8 for fear and disgust experiments, respectively; Figures 4A, B, dotted line).

#### 3.3.2 Emotion induction

Stimulus-evoked EDR frequency during EI in the absence of ingested drugs was significantly lower compared to rest for disgust (*p* = 0.002), but not for fear (*p* = 0.4). Compared to control phase, escitalopram reduced stimulus-evoked EDR frequency for fear (contrast −0.02 ± 0.008 Hz, *p* = 0.021), but not for disgust (*p* = 0.29), while placebo had no effect on any of the two negative emotions (Figures 4A, B, solid line).

#### 3.3.3 Emotion regulation

Stimulus-evoked EDR frequency during ER in the absence of ingested drugs was also significantly lower for disgust compared to rest (*p* < 0.001), but not for fear (*p* = 0.1). Compared to control, escitalopram once again reduced EDR frequency for disgust (contrast −0.1 ± 0.008 Hz, *p* < 0.001) during ER, but not for fear (*p* = 0.95). Placebo had no significant effect on stimulus evoked EDR frequency for any of the two emotions (Figures 4C, D). When psychological and pharmacological emotion regulation were directly compared to each other, we saw no significant differences in stimulus evoked EDR frequency for either fear or disgust (Figure 4E).

### 3.4 Blinding and side-effects of escitalopram and placebo

Roughly 37% of participants guessed the identity of the given substance correctly when given escitalopram, and 13% in the placebo arm wrongly thought they had received escitalopram, while half of the participants either thought they had received placebo or did not want to hazard a guess. The most frequently reported side effects of 10 mg escitalopram were nausea (26%) and fatigue (11%), something that may have aided those who correctly guessed the true identity of the ingested substance. In the placebo arm, fatigue was the most frequently reported side effect (10%) followed by nausea (5%). No information was given in advance as to what effects on the experiments (or otherwise) could be expected from the active substance.

## 4 Discussion

In the present study, we investigated the role of serotonin in the induction and regulation of two negative emotions, fear and disgust, using the selective serotonin reuptake inhibitor (SSRI) escitalopram as a pharmacological probe in a randomized, placebo-controlled, double-blind design and compared this with psychological emotion regulation employing a cognitive strategy with reappraisal. The questions we tried to answer were the following: does single-dose escitalopram have any significant effect on the induction and regulation of the negative emotions, and are these effects similar or different for fear and disgust at the behavioral, cortical, and autonomic levels? Does single-dose escitalopram increase affective tonus at rest (in the absence of emotive stimuli), while at the same time blunting emotional reactivity to emotive stimuli? Does single-dose escitalopram cause emotional dysregulation and render effortful emotion regulation with reappraisal less effective? Is single-dose escitalopram more or less effective in downregulating emotion intensity compared to psychological emotion regulation with reappraisal?

### 4.1 Effects of escitalopram

SSRIs are widely used to treat major depressive and anxiety disorders as well as obsessive-compulsive disorder. It is known that patients started on SSRIs often experience in the early stages of treatment side-effects in the form of increased anxiety, flattened emotion and emotional dysregulation with increased suicidal ideation, an effect known to be strongly age-dependent (36, 37). Based on these observations, we hypothesized that acute ingestion of a therapeutic dose of escitalopram (10 mg) would (i) increase affective tonus (experienced as increased arousal or anxiety) in the absence of emotive stimuli that will be reflected in changes of resting electrodermal activity (EDA), (ii) reduce emotion intensity during emotion induction and (iii) compromise effortful emotion regulation, resulting in dysregulated cognitive control of emotions. All along, we also investigated whether escitalopram had similar or differing effects on the two negative emotions, fear and disgust.

### 4.2 Effects on emotion induction

Escitalopram was associated with decreased emotion intensity of both fear and disgust compared to baseline conditions, confirming our hypothesis that acute ingestion of SSRI leads to emotional blunting. Placebo did not have any significant effect on this, implying that habituation when the tasks were repeated a second time and expectation of medication effect were minimal. Although the role of antidepressants in affective disorders is still debated (25), we nevertheless expected that escitalopram would have greater effect on fear compared to disgust, based on the fact that SSRIs are more effective in treating anxiety related disorders where fear is the core emotion compared to obsessive-compulsive disorders (OCD) where disgust is implicated (6). However, although escitalopram was associated with larger mean reduction in emotion intensity for fear compared to disgust, this difference did not attain statistical significance.

The mechanism through which SSRIs acutely decrease emotion intensity is not known, and both bottom-up and top-down models have been suggested (90). It is possible that this effect might occur at the level of early sensory processing (emotion perception), or cognitive/automatic processing (emotion regulation), or at the level of interoceptive experience (subjective feeling), which all in turn would be expected to affect one way or the other the functional circuits that give rise to emotions. Given the fact that escitalopram had similar effects on both fear and disgust at the behavioral level, i.e., attenuated emotion intensity to similar extents, but had opposite effects on PFC activity, i.e., decreased PFC activation during disgust while increasing it during fear, we draw the conclusion that the similar effects seen at the behavioral level are implemented by different mechanisms in the brain. Direct comparison of the two negative emotions illustrates this contrast more clearly. In a previous study (61), in the absence of any pharmacological intervention, we found greater activation of right ventrolateral PFC during disgust compared to fear, an area known to be involved in response inhibition and in this case possibly reflecting a need for motor inhibition during disgust to suppress emetic impulses (91, 92). However, in the presence of escitalopram, there was instead greater activation in bilateral lateral PFC for fear compared to disgust, with no PFC areas found to be more strongly activated during disgust compared to fear.

This observation is not unique as other studies examining the effects of acute SSRI administration have also demonstrated modulation of PFC activity in different directions (30, 43, 46–48). Jacob and Nienborg (93) theorized that serotonin is implicated early in the process at the feed-forward sensory input stage, modulating the cascade of processes in downstream circuits by affecting the salience of the sensory input or alternatively enhancing network signal/noise efficacy. Although fNIRS does not allow examination of deeper brain areas, studies using fMRI looking at the acute effects of SSRIs have shown attenuated amygdala activity (42, 43, 46), an area with rich sensory input implicated in emotion perception and in coordinating responses to threatening stimuli together with PFC (94). At first glance, this can seem to be inconsistent with reports of increased emotion recognition of fear after SSRI (49, 52, 95) when using experimental designs with emotional facial expressions (46, 52, 95–97). However, the difference between directly presenting emotive stimuli and conveying emotion through facial expression might explain the different results as also illustrated in various studies (98). Presumably, better emotion recognition of facial expressions could depend on the successful transmission of the expressed emotion to the perceiver’s experience (reflecting empathic response), but the opposite is also possible; namely, that less intense own emotional experience would free cognitive resources for correctly identifying the emotion at hand and performing well in the emotion recognition (on someone else) task. As Handley (99) previously suggested, low serotonergic tone may surrender threat responsiveness to brain centers that favor impulsive and automatic responses that lead to increased anxiety while optimizing serotonergic transmission with SSRI might inhibit these areas and enhance flexible and adaptive processing of perceived threat. An often-cited model of emotion regulation postulates increased PFC and decreased amygdala activation in reciprocal manner through top-down and bottom-up pathways, respectively (12, 100, 101). Our results during the fear experiment where PFC activation increased alongside reduced emotion intensity at the behavioral level are compatible with this model.

However, other models of the functional relationship between PFC and limbic areas have also been suggested. For example, conscious labeling of emotions *per se* in the absence of explicit emotion regulation is correlated with increased PFC activation, with inverse relationship to amygdala activation (43, 102–104). It has also been speculated that serotonin might be related to filtering of aversive emotions from coming to conscious awareness (43). The reduction in PFC activation we observed during disgust could reflect a shift of activation in favor of deeper brain areas and associated reduction of conscious awareness of disgust. This is consistent with previous studies (30, 42, 105), where recognition of disgust was reduced after acute SSRI administration. We do not know why this would occur specifically for disgust and not fear, but a possible explanation is that general visceral sensation and emetic reactions ‘diluted’ the stimulus-induced emotional response, reducing the conscious experience of the disgust-pictures themselves.

Thus in agreement with previous studies (46, 47), we found increased PFC activity when viewing fearful stimuli after treatment with single-dose SSRI while the opposite was the case for disgust, indicating that different mechanisms may be at play here. It is worth mentioning that similar to our results, Wolf, Klasen (30) also found reduced PFC activation when subjects viewed aggressive actions that violate social norms, a phenomenon related to moral judgment and assumed to be akin to disgust (106).

Previous studies have also shown physiological differences in the autonomic responses fear and disgust elicit (7, 8, 76, 77). Consistent with this, we found in a previous study (61) significant differences in EDR frequency during emotion induction of fear and disgust, reflecting increased sympathetic activity during fear but not disgust. This difference in EDR frequency between fear and disgust was replicated in the current study in the placebo arm but was abolished by escitalopram. This difference in the autonomic ‘fingerprint’ might be a ‘guiding tool’ utilized for emotion identification when presented with an ambiguous stimulus. In the present study, this ‘fingerprint’ became less distinct after ingestion of escitalopram accompanied by attenuated emotion intensity expressed as flattened subjective feeling.

### 4.3 Effects on psychological emotion regulation

In the absence of any pharmacological intervention, psychological emotion regulation with reappraisal has been shown to reduce emotion intensity by roughly 45% for negative emotions (60, 61). Investigating the role of serotonin on emotion regulation, McRae, Rekshan (90), in a large 8-week long study in patients with major depressive disorder showed that escitalopram improved effortful emotion regulation by helping patients make more use of adaptive strategies like reappraisal, an effect that was significantly correlated with better clinical outcome. Outhred, Das (55), who investigated the acute effects of single dose escitalopram in healthy subjects, similarly concluded that this SSRI facilitated effortful emotion regulation a few hours after its administration. In the present study, we observed no significant effect of escitalopram on emotion regulation with reappraisal, neither for fear nor disgust. More specifically, reappraisal gave a similar reduction (roughly –45%) in emotion rating both in the absence and presence of escitalopram and placebo. We conclude that in our study, contrary to our initial hypothesis, acute ingestion of escitalopram did not lead to dysregulated cognitive control of the negative emotions, and in contrast to what Outhred, Das (55) reported we observed no improved cognitive control of emotion by escitalopram.

### 4.4 Effects on resting affective tonus

Since escitalopram is known to cause increased anxiety as an early side effect in clinical settings (36, 42, 95, 107), we expected that its acute administration would increase resting affective tonus in the absence of emotive stimuli. Previous research (108) has demonstrated increased HPA axis activity and greater secretion of cortisol after acute administration of SSRI. Other researchers (109–111) have found suppression of sympathetic autonomic activity and decrease in noradrenalin plasma levels after repeated SSRI administration. We studied affective tonus using the electrodermal activity (EDA) during rest, namely the non-specific phasic electrodermal response (NS-EDR) frequency, on the assumption that these would increase in frequency reflecting increased arousal (74). In line with this, we found that escitalopram significantly increased resting EDR frequency in the absence of any emotive stimuli during the fear experiment, while placebo had no such effect. However, in the disgust experiment, the effect of escitalopram was smaller and did not reach statistical significance. Our results support previous findings showing increased arousal after acute administration of escitalopram, but it is unclear why this was only prominent in fear and not disgust. A possibility is that there could have been a ‘spillover effect’ of emotive stimuli to the rest period given that fear inducing stimuli are associated with sympathetic activation, while disgust inducing stimuli are not.

### 4.5 Emotion regulation with escitalopram compared to reappraisal

In clinical settings, both pharmacological and psychological interventions are widely used to modify pathological emotional processes. SSRIs as well as cognitive strategies are commonly used within the context of treating depression, anxiety, and obsessive-compulsive disorders, so it is of interest to compare the effects of pharmacological emotion regulation (operationalized as effects of escitalopram on emotion induction) with psychological emotion regulation (operationalized as effortful regulation of emotion with reappraisal). At the behavioral level, we compared the magnitude of emotion reduction by escitalopram with that of reappraisal. Under the conditions of this study, single-session reappraisal was superior to single-dose escitalopram in reducing emotion intensity for both fear (mean change 45% vs. 12%, *p* < 0.001) and disgust (mean change 43% vs. 12%, *p* < 0.001). In OCD, symptoms related to disgust are thought to be more difficult to regulate than symptoms related to anxiety/fear, however emotion regulation with reappraisal has been shown to robustly modify even disgust (112, 113). In an earlier study, we also found that in a non-clinical population, reappraisal was equally effective for fear and disgust (61).

Interestingly, direct comparison of the two emotion regulation strategies on fNIRS activations showed different hemodynamic patterns, with greater activation for escitalopram in right PFC in the fear experiment. According to valence asymmetry hypothesis, the right PFC is associated with withdrawal motivation (70, 114) and processing of negative stimuli (115), but also with down-regulation of emotions (116), which can possibly explain why we observed differences mainly in this area. Furthermore, the two interventions were associated with similar changes of electrodermal activity, but in opposite directions for the two negative emotions. In other words, both interventions altered and blurred the “typical” autonomic profile of the specific emotion seen in the absence of psychological or pharmacological regulation as described above (Figure 4E).

It should be kept in mind, what is being compared here is only the acute effects of these two types of intervention in a non-clinical population. Pharmacotherapy with SSRI and cognitive behavioral therapy (CBT) are both long-term treatments in depression and anxiety disorders. Generally, they are considered to be equally effective in many cases, albeit with some specific differences (22, 23). Responses to SSRI are thought to be dependent on baseline levels of performance and serotonergic function (95, 117, 118), while individuals with anxiety and depression are often found to have decreased prefrontal activation activity during effortful emotion regulation strategies (18, 98, 119).

In a recent study comparing 12 weeks of SSRI or CBT, similar effects between the two interventions were found as well, showing a net effect of reduced amygdala activity during emotion experience and indicating that change in emotion-based markers of brain function did not differentiate the effect of pharmacological and psychological manipulations, rather than differed depending on baseline function and the kind of emotional process (3). The same study showed also considerable variability in change in PFC activation pre-to-post treatment, while in their paradigm they included aversive images of various emotions, implying that the limbic down-regulation could be related to different PFC activation patterns. Although both antidepressants and CBT are thought to modulate the cortico-limbic pathways (65, 120), there is no definite consensus on how SSRIs normalize pathological emotional processing and aide recovery (see also above discussion regarding the effects of escitalopram on emotion induction). Some data support the idea that antidepressants act mainly on the “bottom-up” processes in subcortical emotional networks, leading to a positive bias in the processing of emotional information. Other studies suggest direct engagement with prefrontal regions and a reciprocal negative coupling with the subcortical areas leading to successful emotion regulation (90, 97, 121–123). Cognitive strategies, on the other hand, are thought to affect more directly ‘top-down’ processes leading to increased cognitive control and more adaptive use of regulatory PFC areas (121, 124). It has also been found that psychological treatments that promote cognitive reappraisal skills (3, 62, 63), as well successful psychotherapy improving cognitive control are associated with post-treatment reduction in PFC activity instead (64–66). This observation could explain the lower PFC activation after reappraisal blocks compared to escitalopram, although the former led to great reduction of emotion intensity. When considering the above studies, it is important to bear in mind that single-dose SSRI could yield different, even opposite effects compared to chronic treatment with the same medication (125). Among other things, different pattern of acute versus delayed amygdala response to SSRI has been reported (126).

### 4.6 Conclusions, implications, and future work

We hypothesized increased affective tonus related to single-dose SSRI treatment that would be reflected in changes of autonomic tonus and found that escitalopram but not placebo increased sympathetic activity during rest, but only within the context of the fear experiment. We confirmed our hypothesis regarding acute emotional blunting related to SSRI ingestion, which at the behavioral level was of similar magnitude for both negative emotions. The effects of serotonergic manipulation through administration of SSRIs are multiple and differ depending on the baseline conditions, task at hand and brain area analyzed. Our third hypothesis that escitalopram would cause emotion dysregulation was not confirmed as we did not find any significant effects on the efficiency of reappraisal under the conditions of our study. Lastly, when comparing psychological emotion regulation with pharmacological regulation, at the behavioral level we found greater efficiency for psychological modulation.

These findings imply that the role of serotonin on emotional processing is multifaceted, and it is likely that the effects of SSRI on emotion are more complicated than flattening affect universally. Thus, the variability depending on the individual, their baseline but also the underlying emotion needs to be examined in more detail rather than being considered an epiphenomenon. Future studies could investigate other emotion categories and kinds stimuli as well as explore more on the clinical aspects of this phenomenon in psychiatric cohorts.

## 5 Limitations

The fNIRS signal has low cortical penetration and doesn’t capture hemodynamic changes in deeper cortical areas like the insula or amygdala, which could have added additional information in relation to the PFC activity. The fact that we cannot correlate cortical and subcortical activations using fNIRS imposes limitations on the conclusions we can draw from these experiments. Also, the spatial resolution of fNIRS is low compared to fMRI and although many studies have shown strong correlation between the BOLD and fNIRS signals (67, 68) this still leaves some uncertainty on how well fNIRS can localize cortical activities with the same spatial resolution as fMRI.

## Data availability statement

The original contributions presented in this study are included in the article/Supplementary material, further inquiries can be directed to the corresponding author.

## Ethics statement

The studies involving human participants were reviewed and approved by Stockholm County’s Ethics Committee (Dnr 2013/722-32 and 2014/436-32 and 2022-02605-02). All subjects were given verbal and written information and gave written informed consent through their signature prior to the start of the experiment, in accordance with the Declaration of Helsinki.

## Author contributions

MM conceived this work. MS, MM, and YW contributed to the design and implementation of the research. MS carried out the experiments. MS and YW performed the computations. MS wrote the manuscript with support from MM and YW. All authors contributed to the article and approved the submitted version.

## Footnotes

1
http://www.pstnet.com/eprime.cfm

2
https://github.com/mateusjoffily/EDA/wiki
