# Supplementary material for: Behavioral, cortical and autonomic effects of single-dose escitalopram on the induction and regulation of fear and disgust: Comparison with single-session psychological emotion regulation with reappraisal
Source: Front Psychiatry. 2023 Jan 4;13:988893. doi: 10.3389/fpsyt.2022.988893 (PMC9845894; doi:10.3389/fpsyt.2022.988893)
Supplement: Supplementary file 1 [file Data_Sheet_1.docx]

# Methods

## Emotive stimuli

This study aimed to focus on negative emotions related to clinical diagnosis treated by SSRIs. Furthermore, there is an optimal time window (30-45 min) after which participants may start losing focus and experience discomfort with the fNIRS headgear, which is why the number of emotions studied was limited to two. Two emotions with negative valence and different arousal were therefore chosen, fear and disgust.

The images used in this study, taken from the International Affective Picture System (IAPS) (1), include SAM (Self-Assessment Manikin) scale for valence, arousal, and dominance. They were subsequently tested during a pilot study, where eight individuals rated them for relevance to the emotions we were interested in studying, fear and disgust.

During the present study, we controlled for valence qualitatively and confirmed it was negative for all subjects (as although fear and disgust are considered negatively valenced emotions, theoretically it is possible that they are pleasant for someone).

Subsequently, in our present study, we used the selected images and we also labelled them as inducing ‘fear’ or ‘disgust’ by explicitly giving instructions explaining that ‘pictures planned to be viewed next were chosen for the purpose of inducing the specific emotion’. We assumed therefore, that even if the participants had different internal models and priors regarding what ‘fear’ and ‘disgust’ mean to them, under these controlled conditions the intersubjectivity around these emotional concepts, or as Feldman Barrett (2) has phrased it, by the virtue of collective intentionality, would uniformly influence the trajectory of emotion experience and regulation.

For one of the primary outcomes, the participants were asked to rate the intensity of the induced (or regulated) feeling or emotion. Emotion Induction rating thus, captured arousal (as a physiological and psychological state, and for the former we used EDA as an objective measure as well). The participants were asked to rate the ‘intensity of the experienced (induced or regulated) emotion in response to the image’.

### List of images

**Disgust**: “dirty toilet” (IAPS 9301), “feces” (IAPS 9008), “sewage rat eating rotten meat” (IAPS 1280), “infected gangrenous wound” (IAPS 3266) and “masks crawling in a dirty pond” (IAPS 1111).

**Fear**: “hissing snake” (IAPS 1120), “gaping shark, mouth wide open” (IAPS 1930), “attack pit bull, baring its canines” (IAPS 1300), “spider” (IAPS 1200), and “freshly bleeding hand with severed fingers” (IAPS 3150).

# Results

## Behavioural data

### Variables

| **EI score** | Emotion Induction score (raw in a scale 1-9) | Primary outcome |
| --- | --- | --- |
| **ER score** | Emotion Regulation score (raw in a scale 1-9) | Primary outcome |
| **Psychological Emotion Regulation** | $Psych ER index=\frac{\left( EI in Control - ER in Control \right)}{EI in Control}$ | Secondary outcome** |
| **Pharmacological Emotion Regulation** | $Pharm ER index= \frac{\left( EI in Control - EI after Intervention* \right)}{EI in Control}$ | Secondary outcome** |

* pharmacological intervention with escitalopram or placebo

** secondary exploratory analysis

### Results

| **Behaviour data** |  |  |  |  |  |  |
| --- | --- | --- | --- | --- | --- | --- |
| ***Table 1 (Linear Mixed Models)*** |  |  |  |  |  |  |
| ***Fixed effects*** |  |  |  |  |  |  |
| ***Emotion*** | Fear [0] and Disgust [1] | | | | | |
| ***Condition*** | Emotion Induction [0] and Emotion Regulation [1] | | | | | |
| ***Phase*** | Control [0] and Pharmacological Intervention [1] | | | | | |
| ***Drug*** | Placebo [0] and Escitalopram [1] | | | | | |
| ***Random effects*** | Subject effect, due to repeated measures | | | | | |
|  |  |  |  |  |  |  |
| **Main effects and Interactions** | **Estimate** | **SE** | **z** | **P>\|z\|** | **95% CI (LL)** | **95% CI (UL)** |
| 1.EMOTION | 0.48 | 0.32 | 1.49 | .135 | -0.15 | 1.12 |
| 1.CONDITION | -2.46 | 0.32 | -7.71 | .000 | -3.09 | -1.84 |
| EMOTION#CONDITION | -0.15 | 0.46 | -0.32 | .747 | -1.05 | 0.75 |
| 1.PHASE | -0.12 | 0.32 | -0.37 | .715 | -0.75 | 0.52 |
| EMOTION#PHASE | -0.21 | 0.46 | -0.46 | .645 | -1.12 | 0.70 |
| CONDITION#PHASE | 0.16 | 0.46 | 0.36 | .722 | -0.73 | 1.06 |
| EMOTION#CONDITION#PHASE | 0.00 | 0.66 | 0.00 | .999 | -1.29 | 1.29 |
| 1.DRUG | 0.39 | 0.43 | 0.89 | .373 | -0.46 | 1.24 |
| EMOTION#DRUG | -0.15 | 0.42 | -0.36 | .722 | -0.97 | 0.67 |
| CONDITION#DRUG | -0.03 | 0.41 | -0.08 | .939 | -0.84 | 0.78 |
| EMOTION#CONDITION#DRUG | 0.13 | 0.59 | 0.22 | .827 | -1.03 | 1.29 |
| PHASE#DRUG | -0.65 | 0.42 | -1.54 | .123 | -1.48 | 0.18 |
| EMOTION#PHASE#DRUG | 0.26 | 0.60 | 0.43 | .668 | -0.92 | 1.44 |
| CONDITION#PHASE#DRUG | 0.07 | 0.59 | 0.11 | .911 | -1.10 | 1.23 |
| EMOTION#CONDITION#PHASE#DRUG | 0.11 | 0.85 | 0.13 | .898 | -1.56 | 1.78 |
| Intercept | 5.22 | 0.33 | 15.61 | .000 | 4.57 | 5.88 |
| Wald chi2(15) = 563.99 |  |  |  |  |  |  |
| Prob > chi2 < 0.0001 |  |  |  |  |  |  |
| ICC = 0.54 (SE 0.06) |  |  |  |  |  |  |
| **Contrasts of interest** |  |  |  |  |  |  |
| **Effects of pharmacological intervention** |  |  |  |  |  |  |
| **Disgust** |  |  |  |  |  |  |
| Escitalopram contra Control |  |  |  |  |  |  |
| EI score | -0.68 | 0.28 | -2.41 | 0.016 | -1.23 | -0.13 |
| ER score | -0.36 | 0.28 | -1.28 | 0.201 | -0.91 | 0.19 |
| Placebo contra Control |  |  |  |  |  |  |
| EI score | -0.33 | 0.34 | -0.99 | 0.323 | -0.99 | 0.33 |
| ER score | -0.17 | 0.34 | -0.50 | 0.617 | -0.83 | 0.49 |
| **Fear** |  |  |  |  |  |  |
| Escitalopram contra Control |  |  |  |  |  |  |
| EI score | -0.70 | 0.28 | -2.55 | 0.011 | -1.25 | -0.16 |
| ER score | -0.24 | 0.45 | -0.53 | 0.598 | -1.11 | 0.64 |
| Placebo contra Control |  |  |  |  |  |  |
| EI score | -0.12 | 0.33 | -0.36 | 0.717 | -0.76 | 0.52 |
| ER score | 0.04 | 0.33 | 0.14 | 0.891 | -0.60 | 0.69 |
|  |  |  |  |  |  |  |
| **Emotion regulation:**  **Psychological contra Pharmacological** |  |  |  |  |  |  |
| **Disgust** |  |  |  |  |  |  |
| Escitalopram | -1.85 | 0.28 | -6.55 | <.001 | -2.40 | -1.29 |
| Placebo | -2.28 | 0.34 | -6.78 | <.001 | -2.94 | -1.62 |
| **Fear** |  |  |  |  |  |  |
| Escitalopram | -1.77 | 0.28 | -6.41 | <.001 | -2.31 | -1.23 |
| Placebo | -2.34 | 0.33 | -7.17 | <.001 | -2.99 | -1.70 |

| **Behaviour data** |  |  |  |  |  | |  |  |  |  |
| --- | --- | --- | --- | --- | --- | --- | --- | --- | --- | --- |
| ***Table 2 (paired t-tests)*** | | | | | | | | | | |
| **Escitalopram** |  |  |  |  |  |  | |  |  |  |
|  | **Control** | | | **Intervention** | | | | **Difference** |  |  |
| **Fear** | **n** | **Mean score** | **SD** | **n** | **Mean score** | **SD** | | **SE** | **t** | **p** |
| EI score | 25 | 5.44 | 1.35 | 25 | 4.79 | 1.42 | | 0.15 | 4.25 | <.001 |
| Index Psychological ER | 25 | 0.45 | 0.22 | 25 | 0.46 | 0.22 | | 0.02 | -0.65 | .525 |
|  |  |  |  |  |  |  | |  |  |  |
| **Disgust** |  |  |  |  |  |  | |  |  |  |
| EI score | 24 | 5.85 | 1.49 | 24 | 5.13 | 1.54 | | 0.21 | 3.43 | .002 |
| Index Psychological ER | 24 | 0.43 | 0.23 | 24 | 0.42 | 0.24 | | 0.02 | 0.18 | .860 |
|  |  |  |  |  |  |  | |  |  |  |
| **Placebo** |  |  |  |  |  |  | |  |  |  |
| **Fear** |  |  |  |  |  |  | |  |  |  |
| EI score | 18 | 5.13 | 1.78 | 18 | 5.03 | 1.86 | | 0.24 | 0.43 | .676 |
| Index Psychological ER | 18 | 0.44 | 0.25 | 18 | 0.43 | 0.23 | | 0.02 | 0.35 | .729 |
|  |  |  |  |  |  |  | |  |  |  |
| **Disgust** |  |  |  |  |  |  | |  |  |  |
| EI score | 17 | 5.55 | 1.76 | 17 | 5.22 | 1.77 | | 0.24 | 1.40 | .181 |
| Index Psychological ER | 17 | 0.46 | 0.24 | 17 | 0.46 | 0.23 | | 0.03 | -0.02 | .982 |

## fNIRS

### Variables

| Channels 1-6 | Left prefrontal cortex (LPFC) | Emotion Induction (**EI – Rest)** | Primary outcome |
| --- | --- | --- | --- |
|  |  | Emotion Regulation (**ER – Rest)** | Primary outcome |
| Channels 7-10 | Medial prefrontal cortex (MPFC) | Emotion Induction (**EI – Rest)** | Primary outcome |
|  |  | Emotion Regulation (**ER – Rest)** | Primary outcome |
| Channels 11-16 | Right prefrontal cortex (RPFC) | Emotion Induction (**EI – Rest)** | Primary outcome |
|  |  | Emotion Regulation (**ER – Rest)** | Primary outcome |

### Results

| **fNIRS LPFC data** |  | | | |  |  |  |  |  |
| --- | --- | --- | --- | --- | --- | --- | --- | --- | --- |
| ***Table (Linear Mixed Models)*** |  | | | |  |  |  |  |  |
| ***Fixed effects*** |  | | | |  |  |  |  |  |
| ***Emotion*** | Fear [0] and Disgust [1] | | | | | |  |  |  |
| ***Condition*** | Emotion Induction [0] and Emotion Regulation [1] | | | | | | | | |
| ***Phase*** | Control [0] and Pharmacological Intervention [1] | | | | | | | | |
| ***Drug*** | Placebo [0] and Escitalopram [1] | | | | | | |  |  |
| ***Random effects*** | Subject effect, due to repeated measures | | | | | | | | |
|  |  |  | |  | | |  |  |  |
| **Main effects and Interactions** | **Estimate** | | **SE** | **z** | | | **P>\|z\|** | **95% CI (LL)** | **95% CI (UL)** |
| 1.EMOTION | 0.419 | 0.285 | | 1.47 | | | .141 | -0.139 | 0.977 |
| 1.CONDITION | -0.031 | 0.284 | | -0.11 | | | .912 | -0.588 | 0.525 |
| EMOTION#CONDITION | -0.271 | 0.402 | | -0.68 | | | .499 | -1.058 | 0.516 |
| 1.PHASE | 0.175 | 0.288 | | 0.61 | | | .544 | -0.390 | 0.740 |
| EMOTION#PHASE | -0.511 | 0.405 | | -1.26 | | | .207 | -1.305 | 0.283 |
| CONDITION#PHASE | 0.184 | 0.407 | | 0.45 | | | .651 | -0.614 | 0.982 |
| EMOTION#CONDITION#PHASE | 0.299 | 0.572 | | 0.52 | | | .601 | -0.822 | 1.420 |
| 1.DRUG | 0.127 | 0.290 | | 0.44 | | | .661 | -0.441 | 0.695 |
| EMOTION#DRUG | -0.158 | 0.383 | | -0.41 | | | .681 | -0.908 | 0.593 |
| CONDITION#DRUG | -0.095 | 0.384 | | -0.25 | | | .803 | -0.848 | 0.657 |
| EMOTION#CONDITION#DRUG | 0.215 | 0.540 | | 0.40 | | | .691 | -0.844 | 1.273 |
| PHASE#DRUG | 0.155 | 0.386 | | 0.40 | | | .687 | -0.601 | 0.912 |
| EMOTION#PHASE#DRUG | -0.370 | 0.543 | | -0.68 | | | .496 | -1.434 | 0.694 |
| CONDITION#PHASE#DRUG | -0.259 | 0.544 | | -0.48 | | | .634 | -1.326 | 0.807 |
| EMOTION#CONDITION#PHASE#DRUG | 0.113 | 0.767 | | 0.15 | | | .882 | -1.389 | 1.616 |
| Intercept | 0.175 | 0.215 | | 0.81 | | | .416 | -0.246 | 0.595 |
| Wald chi2(15) = 13.82 |  |  | |  | | |  |  |  |
| Prob > chi2 = 0.5393 |  |  | |  | | |  |  |  |
| ICC = 0.13 (SE 0.05) |  |  | |  | | |  |  |  |
| **Contrasts of interest** |  |  | |  | | |  |  |  |
| **Effects of pharmacological intervention** | | | | | | | | | |
| **Disgust** |  | |  |  | | |  |  |  |
| Escitalopram contra Control |  | |  |  | | |  |  |  |
| EI score | -0.551 | | 0.256 | -2.15 | | | .032 | -1.053 | -0.049 |
| ER score | -0.214 | | 0.256 | -0.83 | | | .404 | -0.716 | 0.288 |
| Placebo contra Control |  | |  |  | | |  |  |  |
| EI score | -0.336 | | 0.285 | -1.18 | | | .238 | -0.894 | 0.222 |
| ER score | 0.147 | | 0.285 | 0.52 | | | .606 | -0.411 | 0.705 |
| **Fear** |  | |  |  | | |  |  |  |
| Escitalopram contra Control |  | |  |  | | |  |  |  |
| EI score | 0.330 | | 0.257 | 1.29 | | | .198 | -0.173 | 0.833 |
| ER score | -0.072 | | 0.291 | -0.25 | | | .804 | -0.643 | 0.499 |
| Placebo contra Control |  | |  |  | | |  |  |  |
| EI score | 0.175 | | 0.288 | 0.61 | | | .544 | -0.390 | 0.740 |
| ER score | 0.359 | | 0.288 | 1.25 | | | .213 | -0.206 | 0.924 |
|  |  | |  |  | | |  |  |  |
| **Emotion regulation: Psychological contra Pharmacological** | | | | | | | | | |
| **Disgust** |  | |  |  | | |  |  |  |
| Escitalopram | 0.367 | | 0.256 | 1.43 | | | .151 | -0.135 | 0.869 |
| Placebo | 0.033 | | 0.285 | 0.12 | | | .906 | -0.524 | 0.591 |
| **Fear** |  | |  |  | | |  |  |  |
| Escitalopram | -0.457 | | 0.257 | -1.78 | | | .075 | -0.960 | 0.046 |
| Placebo | -0.206 | | 0.288 | -0.72 | | | .474 | -0.771 | 0.359 |

| **fNIRS MPFC data** |  | |  |  | |  |  |  |
| --- | --- | --- | --- | --- | --- | --- | --- | --- |
| ***Table (Linear Mixed Models)*** |  | |  |  | |  |  |  |
| ***Fixed effects*** |  | |  |  | |  |  |  |
| ***Emotion*** | | Fear [0] and Disgust [1] | | | | | | |
| ***Condition*** | | Emotion Induction [0] and Emotion Regulation [1] | | | | | | |
| ***Phase*** | | Control [0] and Pharmacological Intervention [1] | | | | | | |
| ***Drug*** | | Placebo [0] and Escitalopram [1] | | | | | | |
| ***Random effects*** | | Subject effect, due to repeated measures | | | | | | |
|  |  | |  | |  |  |  |  |
| **Main effects and Interactions** | **Estimate** | | **SE** | | **z** | **P>\|z\|** | **95% CI (LL)** | **95% CI (UL)** |
| 1.EMOTION | -0.084 | | 0.264 | | -0.32 | .749 | -0.601 | 0.432 |
| 1.CONDITION | -0.246 | | 0.263 | | -0.94 | .349 | -0.761 | 0.269 |
| EMOTION#CONDITION | -0.044 | | 0.372 | | -0.12 | .906 | -0.773 | 0.685 |
| 1.PHASE | -0.317 | | 0.267 | | -1.19 | .235 | -0.840 | 0.206 |
| EMOTION#PHASE | 0.011 | | 0.375 | | 0.03 | .977 | -0.724 | 0.746 |
| CONDITION#PHASE | 0.131 | | 0.377 | | 0.35 | .729 | -0.608 | 0.869 |
| EMOTION#CONDITION#PHASE | 0.398 | | 0.529 | | 0.75 | .452 | -0.639 | 1.435 |
| 1.DRUG | -0.334 | | 0.273 | | -1.22 | .222 | -0.870 | 0.202 |
| EMOTION#DRUG | 0.292 | | 0.355 | | 0.82 | .411 | -0.403 | 0.987 |
| CONDITION#DRUG | 0.153 | | 0.355 | | 0.43 | .667 | -0.543 | 0.849 |
| EMOTION#CONDITION#DRUG | -0.363 | | 0.500 | | -0.73 | .468 | -1.343 | 0.617 |
| PHASE#DRUG | 0.493 | | 0.357 | | 1.38 | .168 | -0.207 | 1.193 |
| EMOTION#PHASE#DRUG | -0.652 | | 0.503 | | -1.30 | .195 | -1.637 | 0.333 |
| CONDITION#PHASE#DRUG | -0.073 | | 0.504 | | -0.15 | .884 | -1.061 | 0.914 |
| EMOTION#CONDITION#PHASE#DRUG | 0.129 | | 0.710 | | 0.18 | .855 | -1.262 | 1.520 |
| Intercept | 0.678 | | 0.203 | | 3.35 | .001 | 0.281 | 1.075 |
| Wald chi2(15) = 15.57 |  | |  | |  |  |  |  |
| Prob > chi2 = 0.4115 |  | |  | |  |  |  |  |
| ICC = 0.16 (SE 0.05) |  | |  | |  |  |  |  |
| **Contrasts of interest** |  | |  | |  |  |  |  |
| **Effects of pharmacological intervention** | | | | | | | | |
| **Disgust** |  | |  |  | |  |  |  |
| Escitalopram contra Control |  | |  |  | |  |  |  |
| EI score | -0.465 | | 0.237 | -1.96 | | .050 | -0.930 | 0.000 |
| ER score | 0.120 | | 0.237 | 0.50 | | .614 | -0.345 | 0.585 |
| Placebo contra Control |  | |  |  | |  |  |  |
| EI score | -0.306 | | 0.264 | -1.16 | | .246 | -0.823 | 0.211 |
| ER score | 0.223 | | 0.264 | 0.84 | | .398 | -0.294 | 0.739 |
| **Fear** |  | |  |  | |  |  |  |
| Escitalopram contra Control |  | |  |  | |  |  |  |
| EI score | 0.176 | | 0.238 | 0.74 | | .459 | -0.290 | 0.642 |
| ER score | 0.239 | | 0.275 | 0.87 | | .385 | -0.300 | 0.778 |
| Placebo contra Control |  | |  |  | |  |  |  |
| EI score | -0.283 | | 0.301 | -0.94 | | .348 | -0.874 | 0.308 |
| ER score | -0.186 | | 0.267 | -0.70 | | .486 | -0.709 | 0.337 |
|  |  | |  |  | |  |  |  |
| **Emotion regulation: Psychological contra Pharmacological** | | | | | | | | |
| **Disgust** |  | |  |  | |  |  |  |
| Escitalopram | -0.035 | | 0.237 | -0.15 | | .883 | -0.500 | 0.430 |
| Placebo | 0.016 | | 0.264 | 0.06 | | .952 | -0.501 | 0.533 |
| **Fear** |  | |  |  | |  |  |  |
| Escitalopram | -0.269 | | 0.238 | -1.13 | | .257 | -0.735 | 0.197 |
| Placebo | 0.071 | | 0.267 | 0.26 | | .791 | -0.452 | 0.593 |

| **fNIRS RPFC data** |  |  |  |  |  |  | |
| --- | --- | --- | --- | --- | --- | --- | --- |
| ***Table (Linear Mixed Models)*** |  |  |  |  |  |  | |
| ***Fixed effects*** |  |  |  |  |  |  | |
| ***Emotion*** | Fear [0] and Disgust [1] | | |  |  |  | |
| ***Condition*** | Emotion Induction [0] and Emotion Regulation [1] | | | | | | |
| ***Phase*** | Control [0] and Pharmacological Intervention [1] | | | | | | |
| ***Drug*** | Placebo [0] and Escitalopram [1] | | | |  |  | |
| ***Random effects*** | Subject effect, due to repeated measures | | | | | | |
|  |  |  |  |  |  | |  |
| **Main effects and Interactions** | **Estimate** | **SE** | **z** | **P>\|z\|** | **95% CI (LL)** | | **95% CI (UL)** |
| 1.EMOTION | 0.431 | 0.287 | 1.50 | .133 | -0.132 | | 0.993 |
| 1.CONDITION | -0.078 | 0.286 | -0.27 | .786 | -0.639 | | 0.483 |
| EMOTION#CONDITION | -0.295 | 0.405 | -0.73 | .466 | -1.089 | | 0.499 |
| 1.PHASE | 0.335 | 0.291 | 1.15 | .248 | -0.234 | | 0.905 |
| EMOTION#PHASE | -0.452 | 0.409 | -1.11 | .269 | -1.252 | | 0.349 |
| CONDITION#PHASE | 0.247 | 0.411 | 0.60 | .548 | -0.558 | | 1.052 |
| EMOTION#CONDITION#PHASE | 0.230 | 0.577 | 0.40 | .690 | -0.900 | | 1.361 |
| 1.DRUG | 0.089 | 0.290 | 0.31 | .759 | -0.479 | | 0.656 |
| EMOTION#DRUG | -0.143 | 0.386 | -0.37 | .711 | -0.900 | | 0.614 |
| CONDITION#DRUG | 0.081 | 0.387 | 0.21 | .833 | -0.677 | | 0.840 |
| EMOTION#CONDITION#DRUG | -0.021 | 0.545 | -0.04 | .969 | -1.089 | | 1.047 |
| PHASE#DRUG | 0.244 | 0.389 | 0.63 | .530 | -0.518 | | 1.007 |
| EMOTION#PHASE#DRUG | -0.437 | 0.548 | -0.80 | .425 | -1.510 | | 0.636 |
| CONDITION#PHASE#DRUG | -0.782 | 0.549 | -1.42 | .154 | -1.858 | | 0.294 |
| EMOTION#CONDITION#PHASE#DRUG | 0.694 | 0.773 | 0.90 | .370 | -0.822 | | 2.210 |
| Intercept | 0.067 | 0.214 | 0.31 | .754 | -0.353 | | 0.487 |
| Wald chi2(15) = 18.06 |  |  |  |  |  | |  |
| Prob > chi2 = 0.2595 |  |  |  |  |  | |  |
| ICC = 0.11 (SE 0.04) |  |  |  |  |  | |  |
| **Contrasts of interest** |  |  |  |  |  | |  |
| **Effects of pharmacological intervention** | | | | | | |  |
| **Disgust** |  |  |  |  |  | |  |
| Escitalopram contra Control |  |  |  |  |  | |  |
| EI score | -0.309 | 0.258 | -1.19 | .232 | -0.815 | | 0.198 |
| ER score | 0.081 | 0.258 | 0.31 | .755 | -0.426 | | 0.587 |
| Placebo contra Control |  |  |  |  |  | |  |
| EI score | -0.116 | 0.287 | -0.40 | .686 | -0.679 | | 0.447 |
| ER score | 0.361 | 0.287 | 1.26 | .209 | -0.202 | | 0.924 |
| **Fear** |  |  |  |  |  | |  |
| Escitalopram contra Control |  |  |  |  |  | |  |
| EI score | 0.580 | 0.259 | 2.24 | .025 | 0.073 | | 1.087 |
| ER score | -0.367 | 0.291 | -1.26 | .207 | -0.938 | | 0.203 |
| Placebo contra Control |  |  |  |  |  | |  |
| EI score | 0.297 | 0.253 | 1.17 | .240 | -0.199 | | 0.793 |
| ER score | 0.582 | 0.291 | 2.00 | .045 | 0.013 | | 1.152 |
|  |  |  |  |  |  | |  |
| **Emotion regulation: Psychological contra Pharmacological** | | | | | | | |
| **Disgust** |  |  |  |  |  |  | |
| Escitalopram | -0.004 | 0.258 | -0.02 | .986 | -0.511 | 0.502 | |
| Placebo | -0.257 | 0.287 | -0.90 | .370 | -0.820 | 0.306 | |
| **Fear** |  |  |  |  |  |  | |
| Escitalopram | -0.576 | 0.259 | -2.23 | .026 | -1.083 | -0.069 | |
| Placebo | -0.413 | 0.291 | -1.42 | .155 | -0.983 | 0.156 | |

| **Pharmacological intervention: Disgust compared to Fear (Emotion Induction)** | | | | | | |
| --- | --- | --- | --- | --- | --- | --- |
|  | **Estimate** | **SE** | **z** | **P>\|z\|** | **95% CI (LL)** | **95% CI (UL)** |
| **Placebo** |  |  |  |  |  |  |
| LPFC | -0.092 | 0.288 | -0.32 | .749 | -0.657 | 0.473 |
| MPFC | -0.074 | 0.267 | -0.28 | .782 | -0.597 | 0.449 |
| RPFC | -0.021 | 0.291 | -0.07 | .943 | -0.590 | 0.549 |
| **Escitalopram** |  |  |  |  |  |  |
| LPFC | -0.620 | 0.256 | -2.42 | .015 | -1.121 | -0.119 |
| MPFC | -0.434 | 0.237 | -1.83 | .067 | -0.898 | 0.030 |
| RPFC | -0.601 | 0.258 | -2.33 | .020 | -1.106 | -0.095 |

## EDA

### Variables

| **EDA during Rest** | EDA frequency (NS EDR/ sec) during all Rest periods | Primary outcome |
| --- | --- | --- |
| **EDA during EI** | EDA frequency (EDR/ sec) during all Emotion Induction blocks adjusted to baseline (EDA Rest) | Primary outcome |
| **EDA during ER** | EDA frequency (EDR/ sec) during all Emotion Regulation blocks adjusted to baseline (EDA Rest) | Primary outcome |
| **Raw EDA EI** | EDA frequency (EDR/ sec) during all Emotion Induction blocks not adjusted to baseline | Secondary outcome * |
| **Raw EDA ER** | EDA frequency (EDR/ sec) during all Emotion Regulation blocks not adjusted to baseline | Secondary outcome * |

*secondary, exploratory analysis

### Results

| **EDA data** |  |  | |  |  |  | |  | | |  |
| --- | --- | --- | --- | --- | --- | --- | --- | --- | --- | --- | --- |
| ***Table (Linear Mixed Models)*** |  |  | |  |  |  | |  | | |  |
| ***Fixed effects*** |  |  | |  |  |  | |  | | |  |
| ***Emotion*** | Fear [0] and Disgust [1] | | |  | | |  | | |  | |
| ***Condition*** | Emotion Induction [0] and Emotion Regulation [1] | | | | | | | | | | |
| ***Phase*** | Control [0] and Pharmacological Intervention [1] | | | | | | | | | | |
| ***Drug*** | Placebo [0] and Escitalopram [1] | | | | | |  | | |  | |
| ***Random effects*** | Subject effect. due to repeated measures | | | | | | | | | | |
|  |  |  |  |  | | |  | |  | | |
| **Main effects and Interactions** | **Estimate** | **SE** | **z** | **P>\|z\|** | | | **95% CI (LL)** | | **95% CI (UL)** | | |
| 1.EMOTION | -0.016 | 0.009 | -1.720 | 0.086 | | | -0.034 | | 0.002 | | |
| 1.CONDITION | 0.091 | 0.009 | 9.750 | < .001 | | | 0.072 | | 0.109 | | |
| EMOTION#CONDITION | 0.032 | 0.013 | 2.420 | 0.016 | | | 0.006 | | 0.057 | | |
| 1.PHASE | 0.002 | 0.009 | 0.220 | 0.822 | | | -0.016 | | 0.020 | | |
| EMOTION#PHASE | 0.001 | 0.013 | 0.100 | 0.924 | | | -0.024 | | 0.027 | | |
| CONDITION#PHASE | -0.102 | 0.013 | -7.780 | < .001 | | | -0.128 | | -0.076 | | |
| EMOTION#CONDITION#PHASE | -0.029 | 0.019 | -1.560 | 0.120 | | | -0.065 | | 0.007 | | |
| 1.DRUG | 0.009 | 0.009 | 1.010 | 0.311 | | | -0.008 | | 0.026 | | |
| EMOTION#DRUG | 0.001 | 0.012 | 0.080 | 0.934 | | | -0.023 | | 0.025 | | |
| CONDITION#DRUG | -0.007 | 0.012 | -0.550 | 0.580 | | | -0.031 | | 0.017 | | |
| EMOTION#CONDITION#DRUG | -0.013 | 0.017 | -0.760 | 0.447 | | | -0.047 | | 0.021 | | |
| PHASE#DRUG | -0.020 | 0.012 | -1.690 | 0.092 | | | -0.044 | | 0.003 | | |
| EMOTION#PHASE#DRUG | 0.025 | 0.017 | 1.460 | 0.143 | | | -0.009 | | 0.059 | | |
| CONDITION#PHASE#DRUG | 0.019 | 0.017 | 1.130 | 0.257 | | | -0.014 | | 0.053 | | |
| EMOTION#CONDITION#PHASE#DRUG | 0.003 | 0.024 | 0.140 | 0.891 | | | -0.044 | | 0.051 | | |
| Intercept | -0.006 | 0.007 | -0.910 | 0.364 | | | -0.019 | | 0.007 | | |
| Wald chi2(15) = 889.78 |  |  |  |  | | |  | |  | | |
| Prob > chi2 < 0.0001 |  |  |  |  | | |  | |  | | |
| ICC = 0.06 (SE 0.04) |  |  |  |  | | |  | |  | | |
| **Contrasts of interest** |  |  |  |  | | |  | |  | | |
| **Effects of pharmacological intervention** |  |  |  |  | | |  | |  | | |
| **Disgust** |  |  |  |  | | |  | |  | | |
| Escitalopram contra Control |  |  |  |  | | |  | |  | | |
| EI score | 0.01 | 0.01 | 1.06 | 0.288 | | | -0.01 | | 0.02 | | |
| ER score | -0.10 | 0.01 | -13.27 | < .001 | | | -0.12 | | -0.09 | | |
| Placebo contra Control |  |  |  |  | | |  | |  | | |
| EI score | 0.00 | 0.01 | 0.37 | 0.714 | | | -0.01 | | 0.02 | | |
| ER score | -0.13 | 0.01 | -14.03 | < .001 | | | -0.15 | | -0.11 | | |
| **Fear** |  |  |  |  | | |  | |  | | |
| Escitalopram contra Control |  |  |  |  | | |  | |  | | |
| EI score | -0.02 | 0.01 | -2.32 | 0.021 | | | -0.03 | | 0.00 | | |
| ER score | 0.00 | 0.01 | -0.06 | 0.954 | | | -0.02 | | 0.02 | | |
| Placebo contra Control |  |  |  |  | | |  | |  | | |
| EI score | 0.00 | 0.01 | 0.23 | 0.819 | | | -0.02 | | 0.02 | | |
| ER score | -0.10 | 0.01 | -10.99 | < .001 | | | -0.12 | | -0.08 | | |

| **Rest EDA data** | |  |  |  |  | |  |  |  |  |
| --- | --- | --- | --- | --- | --- | --- | --- | --- | --- | --- |
| ***Table (paired t-tests)*** | |  |  |  |  | |  |  |  |  |
|  |  |  |  |  |  | |  |  |  |  |
|  | **Control** | | | **Intervention** | | | | **Difference** |  |  |
| **Escitalopram** | **n** | **Mean frq** | **SD** | **n** | | **Mean frq** | **SD** | **SE** | **t** | **p** |
| **Disgust** | 22 | 0.083 | 0.056 | 22 | | 0.083 | 0.071 | 0.012 | -0.06 | 0.954 |
|  |  |  |  |  | |  |  |  |  |  |
| **Fear** | 18 | 0.082 | 0.059 | 18 | | 0.112 | 0.063 | 0.010 | -3.00 | 0.008 |
|  |  |  |  |  | |  |  |  |  |  |
| **Placebo** |  |  |  |  | |  |  |  |  |  |
| **Disgust** | 18 | 0.074 | 0.058 | 18 | | 0.072 | 0.049 | 0.011 | 0.20 | 0.846 |
|  |  |  |  |  | |  |  |  |  |  |
| **Fear** | 15 | 0.095 | 0.048 | 15 | | 0.087 | 0.044 | 0.009 | 0.86 | 0.405 |

| **EDA data (raw)** | |  |  |  |  |  |  |  |  |
| --- | --- | --- | --- | --- | --- | --- | --- | --- | --- |
| ***Table (paired t-tests)*** | |  |  |  |  |  |  |  |  |
| **Escitalopram** |  |  |  |  |  |  |  |  |  |
|  | **Task (EI or ER)** | |  | **Rest** |  |  | **Difference** |  |  |
|  | **n** | **Mean frq** | **SD** | **n** | **Mean frq** | **SD** | **SE** | **t** | **p** |
| **Fear** |  |  |  |  |  |  |  |  |  |
| EI | 21 | 0.084 | 0.066 | 21 | 0.104 | 0.064 | 0.008 | -2.35 | 0.029 |
| ER | 21 | 0.083 | 0.075 | 21 | 0.104 | 0.064 | 0.008 | -2.71 | 0.014 |
|  |  |  |  |  |  |  |  |  |  |
| **Disgust** |  |  |  |  |  |  |  |  |  |
| EI | 22 | 0.078 | 0.068 | 22 | 0.083 | 0.071 | 0.006 | -0.87 | 0.396 |
| ER | 22 | 0.071 | 0.061 | 22 | 0.083 | 0.071 | 0.008 | -1.56 | 0.134 |
|  |  |  |  |  |  |  |  |  |  |
| **Placebo** |  |  |  |  |  |  |  |  |  |
| **Fear** |  |  |  |  |  |  |  |  |  |
| EI | 18 | 0.074 | 0.050 | 18 | 0.079 | 0.046 | 0.006 | -0.82 | 0.423 |
| ER | 18 | 0.061 | 0.043 | 18 | 0.079 | 0.046 | 0.009 | -2.07 | 0.054 |
|  |  |  |  |  |  |  |  |  |  |
| **Disgust** |  |  |  |  |  |  |  |  |  |
| EI | 19 | 0.058 | 0.047 | 19 | 0.078 | 0.053 | 0.005 | -3.63 | 0.002 |
| ER | 19 | 0.049 | 0.042 | 19 | 0.078 | 0.053 | 0.004 | -6.69 | <.001 |

| **EDA data** |  |  |  | | |  |  |  | |  | |  | |
| --- | --- | --- | --- | --- | --- | --- | --- | --- | --- | --- | --- | --- | --- |
| ***Table (paired t-tests)*** |  |  |  | | |  |  |  | |  | |  | |
| **Escitalopram** |  |  |  | | |  |  |  | |  | |  | |
|  |  | **Pharmacological ER*** | | | | **Psychological ER**** | | **Difference** | |  | |  | |
|  | **n** | **Mean frq** | **SD** | | | **Mean frq** | **SD** | **SE** | | **t** | | **p** | |
| **Fear** |  |  |  | | |  |  |  | |  | |  | |
| Escitalopram | 18 | -0.43 | 5.03 | | | -0.56 | 4.42 | 0.86 | | 0.15 | | .885 | |
| Placebo | 15 | 2.00 | 6.48 | | | -5.30 | 20.48 | 6.89 | | 1.06 | | .307 | |
|  |  |  |  | | |  |  |  | |  | |  | |
| **Disgust** |  |  |  | | |  |  |  | |  | |  | |
| Escitalopram | 22 | 0.32 | 3.06 | | | 0.47 | 7.53 | 1.65 | | -0.09 | | .931 | |
| Placebo | 18 | 10.55 | 46.86 | | | 0.80 | 2.44 | 11.11 | | 0.88 | | .392 | |
| * Pharmacological ER = (EDA EI in Control - EDA EI in Intervention phase)/ EDA EI in Control | | | | | | | | | |  | |  | |
| ** Psychological ER = (EDA EI in Control - EDA ER in Control phase)/ EDA EI in Control | | | | | | | | | | |  |  |  |
| (all adjusted to baseline/ EDA Rest) | | | |  |  |  | | |  | |  |  |  |

# Power calculation

An a priori power analysis was conducted using Stata 14 software (StataCorp. 2015. Stata Statistical Software: Release 14. College Station, TX: StataCorp LP) to determine the minimum sample size required to test the study hypotheses. Results indicated the required sample size to achieve 80% power for detecting a medium effect (20% difference between control and intervention), at a significance criterion of α = .05, was N = 20 for paired samples t-tests.

# References

1. Lang PJ, Bradley MM, Cuthbert BN. International affective picture system (IAPS): affective ratings of pictures and instruction manual. Technical Report A-8. 2008;University of Florida, Gainesville, FL.

2. Barrett LF. Emotions Are Real. Emotion (Washington, DC). 2012;12(3):413-29.
